# An early model to predict the risk of gestational diabetes mellitus in the absence of blood examination indexes: application in primary health care centres

**DOI:** 10.1186/s12884-021-04295-2

**Published:** 2021-12-08

**Authors:** Jingyuan Wang, Bohan Lv, Xiujuan Chen, Yueshuai Pan, Kai Chen, Yan Zhang, Qianqian Li, Lili Wei, Yan Liu

**Affiliations:** 1 Department of Respiratory and Critical Care Medicine, The Affiliated Hospital of Qingdao University, Qingdao, China; 2 School of Nursing, Qingdao University, Qingdao, China; 3 Department of Nursing, The Affiliated Hospital of Qingdao University, #16 Jiangsu Road, Qingdao, 266003 Shandong Province China; 4 Department of Critical Care Medicine, The Affiliated Hospital of Qingdao University, Qingdao, China

**Keywords:** Gestational diabetes mellitus, Machine Learning, Prediction model, Maternal and infant health care, Primary health care centre

## Abstract

**Background:**

Gestational diabetes mellitus (GDM) is one of the critical causes of adverse perinatal outcomes. A reliable estimate of GDM in early pregnancy would facilitate intervention plans for maternal and infant health care to prevent the risk of adverse perinatal outcomes. This study aims to build an early model to predict GDM in the first trimester for the primary health care centre.

**Methods:**

Characteristics of pregnant women in the first trimester were collected from eastern China from 2017 to 2019. The univariate analysis was performed using SPSS 23.0 statistical software. Characteristics comparison was applied with Mann-Whitney U test for continuous variables and chi-square test for categorical variables. All analyses were two-sided with *p* < 0.05 indicating statistical significance. The *train_test_split* function in Python was used to split the data set into 70% for training and 30% for test. The Random Forest model and Logistic Regression model in Python were applied to model the training data set. The 10-fold cross-validation was used to assess the model’s performance by the areas under the ROC Curve, diagnostic accuracy, sensitivity, and specificity.

**Results:**

A total of 1,139 pregnant women (186 with GDM) were included in the final data analysis. Significant differences were observed in age (*Z*=−2.693, *p*=0.007), pre-pregnancy BMI (*Z*=−5.502, *p*<0.001), abdomen circumference in the first trimester (*Z*=−6.069, *p*<0.001), gravidity (*Z*=−3.210, *p*=0.001), PCOS (χ^2^=101.024, *p*<0.001), irregular menstruation (χ^2^=6.578, *p*=0.010), and family history of diabetes (χ^2^=15.266, *p*<0.001) between participants with GDM or without GDM. The Random Forest model achieved a higher AUC than the Logistic Regression model (0.777±0.034 vs 0.755±0.032), and had a better discrimination ability of GDM from Non-GDMs (Sensitivity: 0.651±0.087 vs 0.683±0.084, Specificity: 0.813±0.075 vs 0.736±0.087).

**Conclusions:**

This research developed a simple model to predict the risk of GDM using machine learning algorithm based on pre-pregnancy BMI, abdomen circumference in the first trimester, age, PCOS, gravidity, irregular menstruation, and family history of diabetes. The model was easy in operation, and all predictors were easily obtained in the first trimester in primary health care centres.

## Background

Gestational diabetes mellitus (GDM) is a growing public health concern [1–3]. “When hyperglycemia detected during routine testing in pregnancy (generally between 24 and 28 weeks) does not meet the criteria of DIP (either have been pre-existing diabetes antedating pregnancy, or diabetes first diagnosed during pregnancy) it is called GDM” [4]. GDM causes adverse perinatal pregnancy outcomes, such as postpartum haemorrhage, infection, preterm delivery, macrosomia, and neonatal respiratory distress syndrome, and threatens the long-term health of mothers and infants [5–7]. Compared with normal pregnant mothers, women with GDM have a 6–12.6 folds higher risk of developing type 2 diabetes after delivery [8–10]. It is reported that 1 in 4 pregnant women develop T2DM after being diagnosed with GDM, with an average time of about eight years [11]. Moreover, the risk of metabolism-related diseases such as obesity and type 2 diabetes in offspring of women with GDM will also increase significantly [4]. In recent research, it is reported that mothers with GDM have a significantly increased risk of congenital heart defects (CHDs) in offspring (OR = 1.98, 95% CI 1.66–2.36) [8].

With a greater prevalence of obesity and sedentary lifestyles, the global prevalence of GDM has increased from 5.4–7.6% [12, 13] to 14.8–18% [14, 15]. Since the International Association of Diabetes and Pregnancy Study Group (IADPSG) proposed lower diagnostic thresholds [16], the prevalence of GDM has increased further [17, 18]. It is urgent to predict GDM timely and provide intervention strategies to prevent or delay the onset of GDM.

At present, the diagnosis of GDM needs to be confirmed by an Oral Glucose Tolerance Test (OGTT) at the 24th to 28th week of gestation. However, previous studies have found that persistent hyperglycemia during pregnancy can also adversely affect the outcome of a pregnant woman or fetus before a precise diagnosis of gestational diabetes being made [19]. A reliable estimate of GDM in early pregnancy would facilitate intervention plans for maternal and infant health care to prevent the risk of macrosomia, cesarean delivery, etc.

Several models [20–24] have been developed based on a panel of maternal biomarkers consisted of maternal demographics, medical and obstetric histories, and laboratory tests. These models mainly were developed based on at least one blood examination indexes available at the laboratory, such as triglycerides (TG) and HbA1_c_ [20], Prothrombin time (PAT-PT) [21], alanine aminotransferase [22], Lipoprotein(a) [23], and fasting plasma glucose (FPG) [24]. But, in most primary health care centres, the availability of maternal laboratory biomarkers is low, particularly at early pregnancy, due to limited access to laboratory tests for specific blood examination indexes. Although fasting glucose is usually widely available, the utility of first trimester fasting glycemia is limited due to the low accuracy for GDM prediction [24, 25].

This study developed a predictive model for GDM based on maternal demographics, medical histories, and obstetric histories during the first trimester of pregnancy. The proposed model could be implemented in the early stages of pregnancy when maternal laboratory values were not always available in primary health care centres. An earlier prediction of GDM would facilitate intervention plans for maternal and infant health care to prevent the risk of GDM.

## Methods

### Study design

The dataset used in this study was derived from a prospective follow-up cohort of pregnant women established in Qingdao between November 2017 and December 2019. The study was conducted at three primary women and child health care centres and a university-affiliated hospital. The university-affiliated Hospital is a treatment centre for critical and complex cases in eastern China, with 4,500–5,000 deliveries annually. The Medical Ethics Committee of the first author’s university approved the study (Ethical number: QYFYKYLL411311920). All participants were informed of the aims and plan of the study, and written consent was obtained. They were anonymous during the entire research process, and a unified numbering system recorded their identifications.

### Participants

Participants were enrolled in the first trimester (before 14 gestational weeks). The inclusion criteria included women 1) 18 years old and above, 2) who planned to give birth in the study hospital, and 3) who had a singleton pregnancy. Women were not eligible to participate in the study if they: 1) were previously diagnosed with GDM, type I or type II diabetes mellitus, or 2) had cognitive or communication impairments.

### Predictive variables

Baseline maternal characteristics and obstetric histories were prospectively collected in the first trimester (before 14 gestational weeks). Baseline maternal characteristics (height, blood pressure, and abdomen circumference) were measured at enrollment. Age, pre-pregnancy weight and obstetric histories (gravidity, parity, obstetric abnormality, polycystic ovary syndrome (PCOS), irregular menstruation, and family history of diabetes) were collected through face-to-face interviews with self-completed questionnaires. The pre-pregnancy BMI was calculated using the measured height and the self-reported pre-pregnancy weight. The gravidity refers to the number of pregnancies a participant has had since puberty, and the parity refers to the number of times a participant has given birth. The obstetric abnormality recorded history of abnormal gravidity (preterm birth, miscarriage and induced abortion). Women were asked about the interval of two menstrual cycles in the last 12 months, and irregular menstruation was marked if a menstrual cycle was < 21 days or > 35 days, or a menstruation period lasted < 2 days or > 7 days.

The diagnosis of GDM was based on results of a one-step 2–h 75–g OGTT test administered at the 24th to 28th week of gestation, according to the IADPSG criteria. Participants whose blood glucose levels at fasting, 1–h, or 2–h after taking sugar reached or exceeded 5.1, 10.0, and 8.5mmol/L [25], respectively, were diagnosed as GDM.

### Statistical analysis

The collected data were input into Excel 2016, and all the categorical variables were processed as 0/1 variables. The output variable was predicted by whether GDM was diagnosed at the 24th to 28th week of gestation. If GDM was diagnosed, the result was marked as 1, and if the OGTT was normal, it was marked as 0. The univariate analyses were performed using SPSS 23.0 statistical software (SPSS Inc., Chicago, IL, USA). Continuous data were presented as the mean ± standard deviation, and categorical variables were presented as frequencies (percentages). Characteristics comparison between women with or without GDM was applied with Mann-Whitney U test (all continuous variables were non-normal distribution parameters) for continuous variables and chi-square test for categorical variables. All analyses were two-sided with *p* < 0.05 indicating statistical significance.

#### Prediction methods and model evaluation

The *train_test_split* function of the sklearn package in Python (version 3.8.5) was used to split the data set into 70% as the training data set and 30% as the test data set [26]. Firstly, all the variables were used to develop the prediction model. Then, variables with a *p* value less than 0.05 in the univariate analysis were used to develop the prediction model. The Random Forest (RF) model and Logistic Regression model in Python (version 3.8.5) were applied to model the training data set. The parameters settings of the Random Forest model were a maximum tree depth of none, the number of trees fixed at 100, and the gini splitting criterion. The feature importance function of the Random Forest model was used to rank the importance of variables.

The roc_curve function of the sklearn package was used for the Receiver Operating Characteristic Curve (ROC) analysis on the test data set. The 10-fold cross-validation was used to assess the predictive accuracy of the Random Forest model and the Logistic Regression model by the areas under the ROC Curve (AUC), diagnostic accuracy, sensitivity, and specificity. When ordinary gestation women in the test set were predicted to be normal gestation pregnancies by the model, it was marked as a True Negative (TN). Otherwise, when normal gestation pregnancies were predicted to be GDM patients, it was marked as a False Positive (FP). Similarly, when GDM patients in the test set were predicted to be normal by the model, it was marked as a False Negative (FN). Conversely, when GDM patients were correctly predicted to be GDM patients, the result was marked as a True Positive (TP). Diagnostic accuracy was defined as the proportion of all participants who were correctly predicted by the model (Accuracy = (*TP* + *TN*)/(*FP* + *TN* + *TP* + *FN*). Sensitivity was defined as the percentage of GDM patients whose GDM status was successfully detected (Sensitivity = *TP*/(*TP* + *FN*)). Specificity was defined as the proportion of normal gestations that was successfully detected (Specificity = *TN*/(*TN* + *FP*)).

## Results

### Baseline characteristics

A total of 1,139 pregnant women were included in the final data analysis, and the incidence of GDM diagnosed at the 24th to 28th week of gestation was 16.33% (186/1139). Comparing with participants who did not have GDM, participants with GDM were older (*Z*=−2.693, *p*=0.007), and had higher pre-pregnancy BMI (*Z*=−5.502, *p*<0.001), abdomen circumference in the first trimester (*Z*=−6.069, *p*<0.001), and gravidity (*Z*=−3.210, *p*=0.001) (Table 1). Similarly, participants with GDM had a higher proportion of PCOS (χ^2^=101.024, *p*<0.001), irregular menstruation (χ^2^=6.578, *p*=0.010), and family history of diabetes (χ^2^=15.266, *p*<0.001) (Table 1). There were no significant differences in height, Systolic pressure (SBP), Diastolic pressure (DBP), number of parity, or gestational week at inclusion between the two groups. The proportion of obstetric abnormality was not different between the two groups.

**Table 1:** Baseline maternal characteristics (before14 gestational weeks) among participants with or without GDM

		Non-GDM (*n*=953) mean (SD)/*n*(%)	GDM (*n*=186) mean (SD)/*n*(%)	*Z*/*χ*^*2*^	*p*
Age,(year)		31.10(4.35)	32.22(4.77)	-2.693	0.007
Height,(cm)		163.04(4.52)	163.11(4.73)	-0.572	<0.568
Pre-pregnancy BMI,(kg/m^2^)		21.21(2.51)	22.62(3.38)	-5.502	<0.001
Abdomen circumference at the first trimester,(cm)		79.08(6.06)	82.63(7.63)	-6.096	<0.001
Systolic pressure,(mmHg)		115.06(10.18)	115.51(10.40)	-1.087	0.277
Diastolic pressure,(mmHg)		71.17(7.11)	71.43(7.46)	-0.620	0.535
Gravidity		1.88(1.00)	2.14(1.08)	-3.210	0.001
Parity		0.47(0.52)	0.54(0.55)	-1.415	0.157
Obstetric abnormality	Yes	357(37.46)	80(43.01)	2.027	0.154
	No	596(62.54)	106(56.99)		
Polycystic ovary syndrome	Yes	55(5.77)	55(29.57)	101.024	<0.001
	No	898(94.23)	131(70.43)		
Irregular menstruation	Yes	746(78.28)	161(86.56)	6.578	0.010
	No	207(21.72)	25(13.44)		
Family history of diabetes	Yes	189(19.83)	61(32.80)	15.266	<0.001
	No	764(80.17)	125(67.20)		
Gestational week at inclusion		15.41(2.99)	15.38(3.80)	-0.769	0.442

### Performance in predicting gestational diabetes mellitus risk

**Fig. 1:**
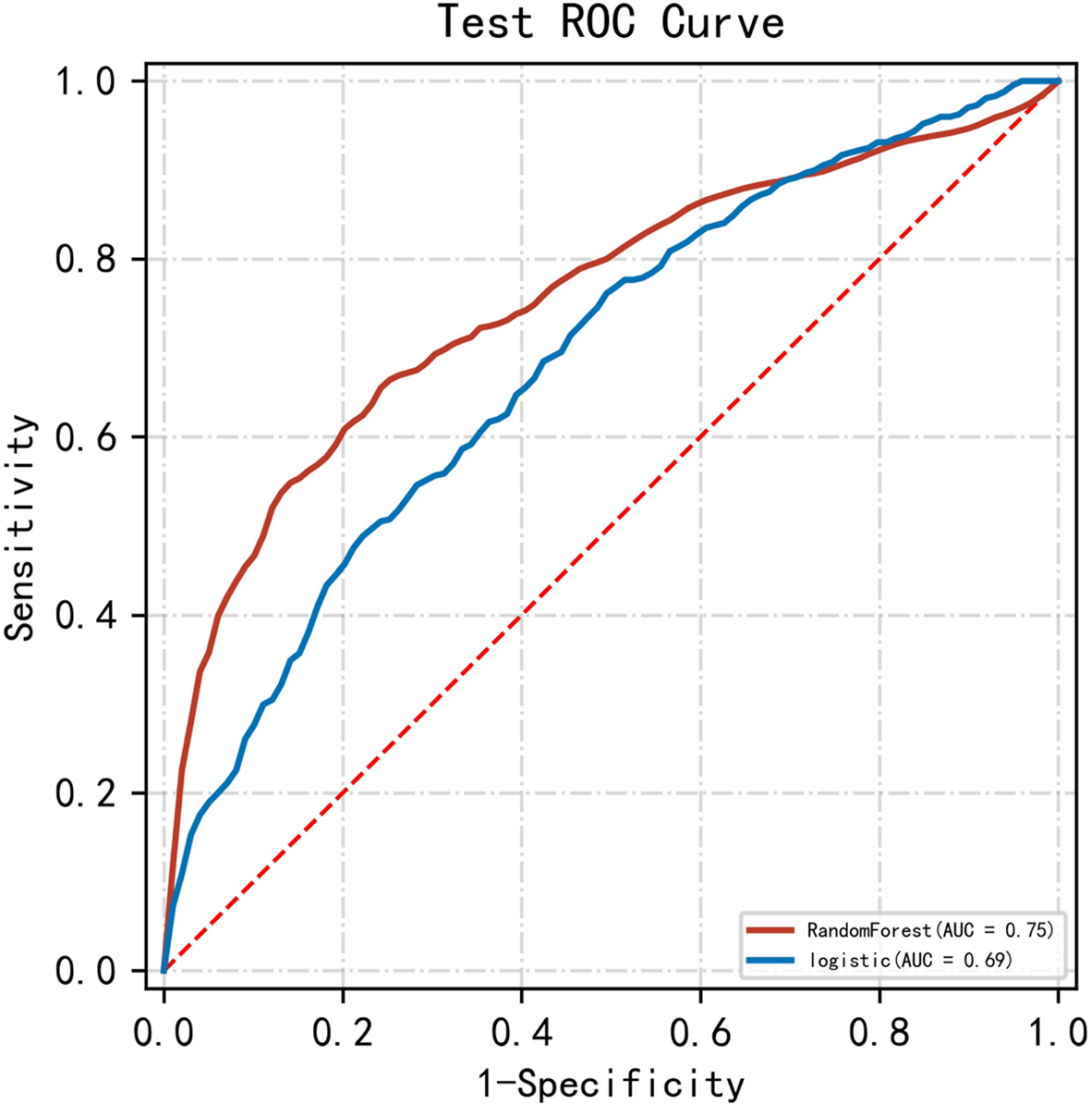
Receiver operating characteristic curve for estimating the discrimination of the Random Forest Model and the Logistic Regression model with all the variables. AUC, the area under the receiver operating characteristic curve

Firstly, all the variables (Table 1) were used to develop the prediction model. The performances of the Random Forest model and the Logistic Regression model were evaluated with the ROC curve and the AUC score (Fig. 1). The 10-fold cross-validation results of the two models are demonstrated in Table 2.

**Table 2:** Performances of the random forest model and the logistic regression model for the prediction of GDM with all the variables

	Model	AUC	Accuracy	Sensitivity	Specificity
Mean	RF	0.754	0.759	0.695	0.764
SD	RF	0.049	0.092	0.132	0.130
Mean	LR	0.686	0.655	0.679	0.656
SD	LR	0.046	0.100	0.188	0.155

*Abbreviations: RF* Random Forest, *LR* Logistic Regression, *AUC* area under the receiver operating characteristic curve

**Fig. 2:**
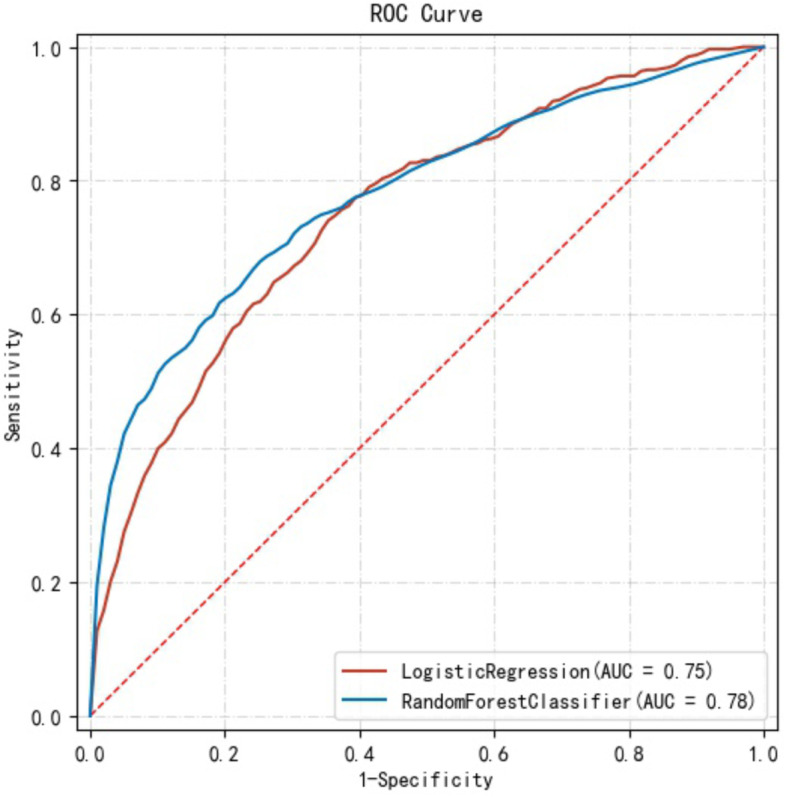
Both the Random Forest Model and the Logistic Regression model had better performance in the ROC curve and AUC after dimensionality reduction. Abbreviations:  AUC, the area under the receiver operating characteristic curve

**Fig. 3:**
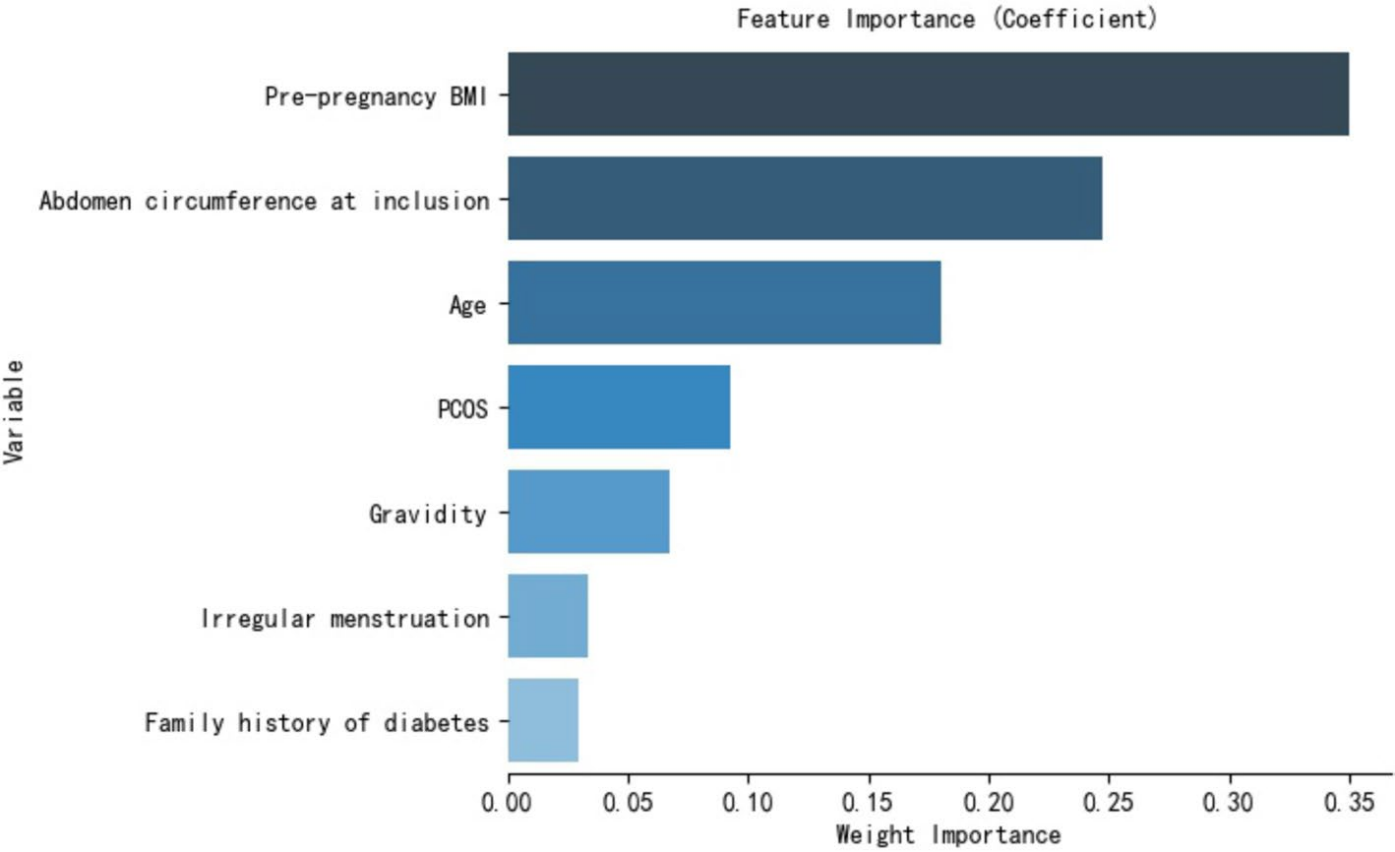
Feature importances of variables in the Random Forest Model

Then, variables that were statistically significantly associated with GDM in univariate analysis were used to develop the prediction model. It included age, pre-pregnancy BMI, abdomen circumference in the first trimester, gravidity, PCOS, irregular menstruation and family history of diabetes. The data dimensionality reduction improved both the two model’s performances (Fig. 2 and Table 3). Feature importance analysis showed that pre-pregnancy BMI was the most important risk factor contributing to GDM events, followed by abdomen circumference in the first trimester of pregnancy, age, PCOS, gravidity, irregular menstruation and family history of diabetes (Fig. 3).

**Table 3:** Both the random forest model and the logistic regression model had better performance after dimensionality reduction

	Model	AUC	Accuracy	Sensitivity	Specificity
Mean	RF	0.777	0.789	0.651	0.813
SD	RF	0.034	0.052	0.087	0.075
Mean	LR	0.755	0.724	0.683	0.736
SD	LR	0.032	0.065	0.084	0.087

*Abbreviations: RF* Random Forest, *LR* Logistic Regression, *AUC* area under the receiver operating characteristic curve

## Discussion

Gestational diabetes mellitus (GDM) is a condition that eludes a single etiology. Early prediction and intervention are essential to achieve the best perinatal outcome and improve maternal and infant health care. Machine learning approaches had the potential to be used to achieve such early predictions. This study developed and validated a Random Forest model for the prediction of GDM. It could be used as an early predictive model of GDM for the primary health care centre based on several simple variables without blood examination indexes. The model incorporated seven risk factors including pre-pregnancy BMI, age, abdomen circumference in the first trimester, gravidity, PCOS, irregular menstruation, and family history of diabetes.

The association of pre-pregnancy BMI with GDM had been explored [27–29]. Compared with the women with normal BMI, obese women had a 3.98-fold risk of developing GDM, and the risk of GDM increased linearly with maternal BMI [27]. In this study, pre-pregnancy BMI was higher in participants with GDM than in participants without GDM. The feature importance was an indicator in Random Forest that marked the contribution of a variable to distinguish cases with GDMs from normal ones. Feature importance analysis showed that pre-pregnancy BMI occupied the highest weight in the Random Forest model. These findings implicated pre-pregnancy BMI as the potential index to distinguish between women with GDM and normal ones. Similar to pre-pregnancy BMI, this study observed significant differences in age, abdomen circumference in the first trimester, gravidity, PCOS, irregular menstruation, and family history of diabetes between women with or without GDM. These results were consistent with previous studies [15, 30–34] and suggested that abdomen circumference in the first trimester, age, PCOS, gravidity, irregular menstruation, and family history of diabetes were potential predictors of GDM. The feature selection and data dimensionality reduction improved the performance of the Random Forest model and the Logistic Regression model.

ROC curve is a comprehensive index that graphically reflects the relationship between sensitivity and specificity. The higher the sensitivity, the fewer missed cases, and the lower the 1-specificity, the less the misdiagnosis rate. The point closest to the upper left corner of a ROC curve is a boundary value. At this point, the sensitivity and specificity are high, and the false positive and false negative are the least. If there are two ROC curves, the curve closer to the upper left corner has a better diagnostic value and a larger AUC. In this study, the ROC curve of the Random Forest model was closer to the upper left corner than the Logistic Regression model, so it achieved a better performance in prediction.

In this study, the Random Forest model achieved an acceptable AUC, which was as high as 0.777±0.034, and had a good discrimination ability for GDM (Sensitivity: 0.651±0.087, Specificity: 0.813±0.075). The performance was similar to that of a recent study [22]. The study developed an XGBoost model for GDM that showed moderate methodological quality with an AUC of 0.742 (95%CI, 0.715-0.769), a median sensitivity of 0.616 and a specificity of 0.769. As mentioned previous, the sensitivity of the model represented the proportion of GDM patients who were successfully identified. The higher the sensitivity, the lower the missed diagnosis rate of GDM patients. The FP rate (1 – Specificity) referred to the proportion of normal individuals misdiagnosed as GDM. In general, an ideal model is characterized by the combination of high sensitivity and low FP rate. So, compared with XGBoost model in the previous literature, the Random Forest model in this study achieved higher sensitivity and fewer FP rate for classifying pregnant women at risk for GDM.

Various machine learning models have been proposed to predict the risk of GDM based on various variables [20–24]. However, these models were not commonly used in the primary health care centre. First, this could be partly attributed to the poor sensitivity of these models since retrospective data with high heterogeneity were often used during their development. Secondly, indicators, such as triglycerides (TG) and HbA1_c_, Prothrombin time (PAT-PT), alanine aminotransferase, and Lipoprotein(a), yield a high AUC, but also increase the difficulty to access, particularly at early pregnancy. The model developed in this study contained seven easily obtained indicators, which would not increase the psychological and economic burden, and was especially suitable for primary health care centres.

In conclusion, using the machine learning method on pre-pregnancy BMI, abdomen circumference in the first trimester, age, PCOS, gravidity, irregular menstruation, and family history of diabetes could distinguish women with GDM and normal ones. The point was that obtaining these indexes in the first trimester of gestation was a simple and inexpensive activity, especially true in primary health care centres where laboratory tests for specific blood examination indexes were not always available.

The strengths of this study included simple indexes from the first trimester of gestation and a population-based prospective data set. The study accurately obtained the abdominal circumference data in the first trimester and minimized the recalling bias of pre-pregnancy weight. This study had the following limitations. First, the general applicability of the prediction model was limited by data derived from a single region. Second, the prospective cohort of pregnant women in primary health care centres reached a moderate sample size. However, the final sample size (n=1139) was sufficient for establishing the machine learning model. To improve the model’s generalizability in future studies, the authors plan to expand the cohort to include additional sampling sites and a more significant number of pregnant women and to use additional data for external verification.

## Conclusions

This research developed a simple model to predict the risk of GDM using machine learning algorithm in the first trimester without blood examination indexes. Predictors including pre-pregnancy BMI, abdomen circumference in the first trimester, age, PCOS, gravidity, irregular menstruation, and family history of diabetes were easily obtained in the first trimester in the primary health care centre. The model was easily used and would facilitate intervention plans for maternal and infant health care to prevent the risk of GDM in early pregnancy.

## Data Availability

The datasets used and analyzed during the current study are available from the corresponding author on reasonable request.
